# Neuropsychological management of multiple sclerosis: evaluation of a supervised and customized cognitive rehabilitation program for self-used at home (SEPIA): protocol for a randomized controlled trial

**DOI:** 10.1186/s13063-019-3715-7

**Published:** 2019-10-29

**Authors:** Caroline Harand, France Daniel, Audrey Mondou, Damien Chevanne, Christian Creveuil, Gilles Defer

**Affiliations:** 1 MS Expert Centre, Department of Neurology, Caen University Hospital Centre, Avenue de la Côte de Nacre CS 30001, 14033 Caen, Cedex 9 France; 2 Réseau Bas-Normand de prise en charge des patients atteints de SEP, 29 rue du Général Moulin, 14000 Caen, France; 3 Biostatistics and Clinical Research Unit, Caen University Hospital Centre, Avenue de la Côte de Nacre CS 30001, 14033 Caen, cedex 9 France

**Keywords:** Cognitive rehabilitation, Quality of life, Self-esteem, Multiple sclerosis, Randomized controlled trial

## Abstract

**Background:**

Cognitive and mood disorders negatively impact daily life in patients with multiple sclerosis (MS). Pharmacological treatments did not demonstrate any effect on cognition compared with cognitive rehabilitation (CR). However, if CR programs offer promising results on cognition, they are less consistent concerning mood and quality of life (QoL). In this context, we designed a randomized controlled trial to evaluate the efficacy of an innovative computerized CR program, conducted at home, on QoL. Secondary objectives will estimate the improvement, or the stabilization over time, of patients’ cognitive performances and their emotional affects.

**Methods:**

Forty MS patients (relapsing-remitting or secondary progressive forms) who have cognitive impairment will be recruited for the trial (called SEPIA-NCT03471338) and randomly assigned to either the experimental group or the control group. Patients randomly assigned in the experimental group will perform a home-based CR program with psychological support during eight consecutive weeks. CR will be based on computerized cognitive exercises from the PRESCO® software developed by HAPPYneuron^©^. Training sessions (three sessions of 45 min per week) will consist of short exercises evaluating a broad range of cognitive domains and will be personalized for each patient (tracking tool and supervised guidance). The control group, designed to control for non-specific elements of the intervention, will receive only psychological support consisting of various issues related to MS, such as everyday cognitive-related difficulties or management of emotions. QoL, assessed by the MUSIQOL (Multiple Sclerosis International Quality Of Life) questionnaire, will be evaluated three times (at baseline and after 1 week and 25 weeks after home-based intervention) as well as secondary outcomes measuring self-esteem, cognition, depression, anxiety, metacognition, fatigue, and sleep quality. Given the expected MUSIQOL variation, the inclusion of 20 patients per group (alpha risk 5% and power 80%) will be required.

**Discussion:**

Evidence suggests that computerized programs may be a practice option for CR for people with MS, but there is a paucity of studies evaluating QoL. We hope that this innovative program will highlight such benefits over time in patients’ daily life. In the future, such programs will allow a wider range of available therapeutic options for MS patients with cognitive impairment and for practitioners in charge of their care.

**Trial registration:**

ClinicalTrials.gov identifier: NCT03471338. Retrospectively registered on 25 April 2018. https://clinicaltrials.gov/ct2/show/NCT03471338?term=NCT03471338&cond=Multiple+Sclerosis&draw=2&rank=1.

## Background

The existence of cognitive disorders in multiple sclerosis (MS) has been widely studied over the last 20 years [1, 2]. These disorders may be considered the first predictive factor of high occupational disability rates [3] and have significant consequences for social, familial, and professional life (e.g., difficulties in meeting the demands of a job, resulting in dismissal, reclassification, or unemployment [3]).

Objective cognitive disorders, detected through traditional neuropsychological assessments, correlate weakly with cognitive complaints reported by patients [4]. In addition, current cognitive assessments do not capture real-world consequences of these deficits as reported by the patients [2]. The current challenge therefore is to find and offer effective alternatives to help patients managing their cognitive problems in daily life [3]. Cognitive rehabilitation (CR) may represent an alternative approach as it does not imply adverse side effects compared with pharmacological interventions [5].

Computer-assisted techniques are among the CR evidence-based practice options for health professionals, supplying a range of benefits [6]. They offer self-paced, individualized training providing new and challenging exercises [3, 7]. The level of difficulty of the task can be changed on the basis of the patient’s baseline skills and can be gradually adjusted as performance improves. They allow a feasible, simple, and friendly activity that requires less face-to-face intervention of health professionals in charge of CR [3, 5]. Stuifbergen et al. conducted an at-home computer-based CR trial in order to help patients with MS to achieve the highest level of cognitive functioning and functional independence [3]. The authors showed that the intervention group outperformed the control group on all measures, including a better personal efficiency, more frequent use of compensatory strategies, and improved performance in neuropsychological tests [3]. In addition, Messinis et al. reported improved verbal and visuospatial episodic memory, information processing speed, and executive functioning after an intervention of 20 individualized 1-hour sessions over a 10-week period using RehaCom® (HASOMED GmbH, Magdeburg, Germany) software in relapsing-remitting (RR) MS patients with mild to moderate cognitive disorders [8]. A systematic review identified a paucity of studies, including activities of daily living as secondary outcomes, among other criteria, with promising results [6].

However, as evidenced by two recent Cochrane reviews [9, 10] and a systematic literature review of 33 studies [11], the efficacy of rehabilitation techniques remains weak and inconclusive. In fact, most of them involved methodological flaws such as time of intervention, goals, frequency of sessions, and content as well as underlying theoretical bases. Further work is required to demonstrate the potential long-term effect of CR. Better outcome measurements and related ecological validity are also warranted. Finally, rehabilitation studies have used two main approaches: those aimed at increasing cognitive performance [6] and those that adopt a holistic approach [12], aiming to improve quality of life (QoL). Although most studies emphasize the need to consider QoL, they sparsely consider this field as a primary outcome. Similar conclusions can be drawn for other concepts such as self-esteem.

Despite these promising results, no computer-assisted rehabilitation trial has focused on improving QoL or mood disorders to date. Most studies aimed to improve cognitive functioning through increased performance during neuropsychological testing. However, these assessments are lacking ecological sensitivity, and potential improvements include more than a cognitive score but rather cover various aspects of our psychic functioning. According to this overview, qualitative, more holistic approaches must be conducted to better evaluate the effect of CR on patient self-perception. Previously, we had a first experience on 10 MS patients with cognitive impairment during an open non-controlled trial using a similar CR home-based program. This pilot study (unpublished) showed that patients get some clinical benefit (based on self-perception questionnaire) in their QoL.

Therefore, we designed this trial in order to compare the effect of an innovative home-based program of computerized CR with psychological support versus a control psychological intervention on QoL in MS patients presenting cognitive impairment. In addition, we will estimate the improvement or stabilization over time of self-esteem, cognitive performance, emotional affect (depression and anxiety), metacognition, fatigue, and sleep quality as secondary measure outcomes which represent important issues for clinical care of patients with MS.

## Methods

### Design

**Fig. 1:**
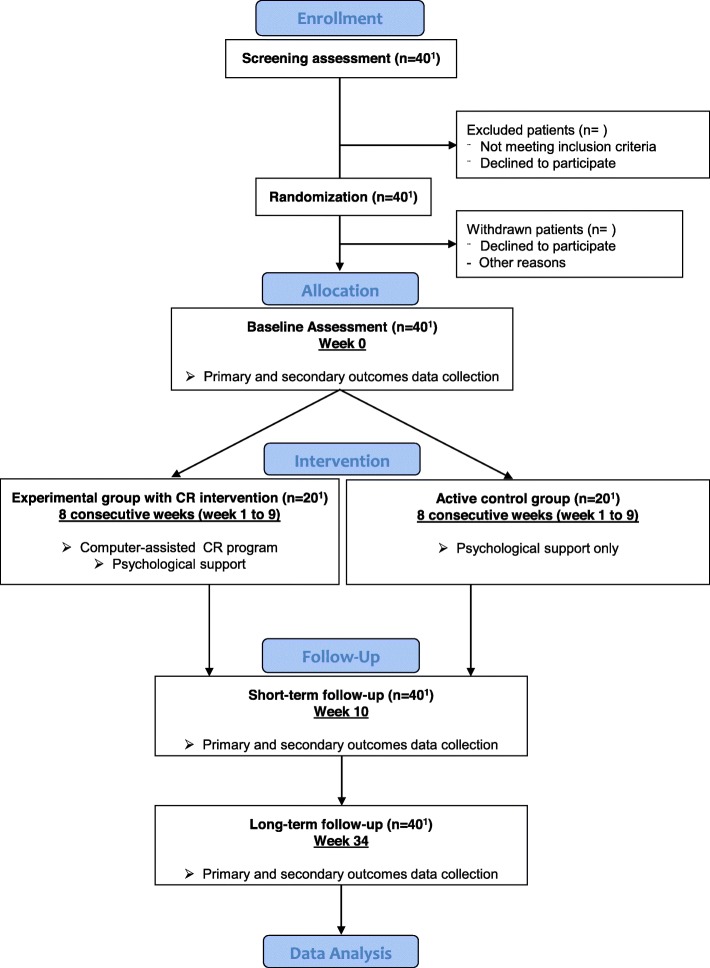
Flowchart of the trial design. *Abbreviation*: *CR* cognitive rehabilitation. ^1^Number of planned patients to recruit

This study is a randomized controlled trial with three data collection points (Fig. 1): baseline (week 0), short-term retest (week 10) one week after the end of the home-based intervention (8 consecutive weeks), and long-term retest at week 34. The trial was registered in ClinicalTrials.gov registry as NCT03471338. A Standard Protocol Items: Recommendations for Interventional Trials (SPIRIT) checklist is provided in Additional file 1.

### Site and participant recruitment

The trial will be monocentric and conducted at the MS Expert Centre, Department of Neurology, Caen University Hospital, Normandy, France. The CR program (cognitive exercises and psychological support) will be performed at patients’ homes in Western Normandy.

Volunteers will be identified during routine care in the outpatient clinic of the MS Expert Centre. Verbal and written detailed information about the ongoing study will be provided to the patient in a standardized way by using a specific informed consent form that has been validated by the ethics committee. Recruitment will take place over the course of 30 months.

### Ethical approval

This trial was registered under “Minimal risk and constraint interventional research” in accordance with the terms of article L.1121–1 of the French Public Health Code (act number 2012–300 of March 5, 2012, and its implementing decree number 2016–1537 of November 16, 2016). Ethical approval was granted by the French Regional Ethics Committee “CPP Nord Ouest II” on September 7, 2017 (ID RCB 2017-A017 36–47).

### Participants

To be eligible for participation, subjects must meet the following defined inclusion criteria:

- confirmed diagnosis of MS in accordance with the 2010 revised McDonald criteria [13]
- RR or secondary progressive (SP) phenotypes of the disease
- be male or female between 18 and 65 years of age
- disease duration of not more than 25 years
- Expanded Disability Status Scale score (EDSS) of not more than 5.5
- clinically inactive phase of the disease as defined by the new Lublin criteria [14]
- impaired cognitive performance at least 1.65 standard deviations below normative data at one test of the BCcogSEP battery [15]
- French native speaking
- computer with Internet access holder
- legal capacity to consent to the trial (via written informed consent).

Exclusion criteria will be as follows:

- other neurological, psychiatric, or developmental diseases prior to MS diagnosis
- head injury sequelae
- chronic alcohol or drug consumption
- EDSS score of at least 6
- presence of relapse or treatment with corticosteroids at least 1 month before recruitment (or both)
- patient under wardship
- cognitive assessment within the last 6 months (particularly including all or some of the tests proposed in the present trial)
- presence of dementia in accordance with the criteria of the fifth edition of the *Diagnostic and Statistical Manual of Mental Disorders* (DSM V) [16] or sufficient cognitive impairment that may interfere with cognitive assessment or the administration of CR exercises (or both)
- any visual or motor deficit that may prevent cognitive assessments or CR exercise administration (or both).

#### Exclusion procedure

Intercurrent disease diagnosed during follow-up, occurrence of relapses (whether or not treated with corticosteroids), patient’s decision or investigator’s decision (or both) for any reason will result in withdrawal of the assigned treatment. However, outcome data will be collected on those patients and included in the intention-to-treat analysis.

### Outcome measurements

For an overview of outcome assessment timelines, see Table 1.

**Table 1:** Schedule of assessments

		Study period												
		Screening	Baseline	Home-based intervention									Short-term follow-up	Long-term follow-up
Visits (weeks)		W-1	W0	W1	W2	W3	W4	W5	W6	W7	W8	W9	W10	W34
Informed consent		X												
Eligibility screen		X												
Randomization		X												
Neuropsychological assessment 1	Cognitive complaint questionnaire	X												
	BCcogSEP	X												
	VAPS	X												
	Multiple errands test	X												
Neuropsychological assessment 2	MUSIQOL		X										X	X
	SEI		X										X	X
	MADRS		X										X	X
	HAMA		X										X	X
	BICAMS = SDMT, CVLT-II, BVMT-R		X										X	X
	MCQ-30		X										X	X
	EMIF-SEP		X										X	X
	PSQI		X										X	X
Active control group	At-home set-up			X										
	Phone follow-up				X	X	X		X	X	X	X		
	At-home follow-up							X						
Experimental group with CR intervention	CR intervention				X	X	X	X	X	X	X	X		
	At-home set-up			X										
	Phone follow-up				X	X	X		X	X	X	X		
	At-home follow-up							X						

*Abbreviations*: *BCcogSEP* Batterie Courte d’évaluation des fonctions cognitives destinées aux patients souffrant de Sclérose En Plaques (French brief test battery evaluating cognitive functions in multiple sclerosis), *BICAMS* Brief International Cognitive Assessment for Multiple Sclerosis, *BVMT-R* Brief Visuospatial Memory Test – Revised, *CR* Cognitive rehabilitation, *CVLT* California Verbal Learning Test, *EMIF-SEP* Echelle Modifiée d’Impact de la Fatigue dans la Sclérose En Plaques (modified fatigue impact scale – multiple sclerosis), *HAMA* HAMilton Anxiety rating scale, *MADRS* Montgomery and Åsberg Depression Rating Scale, *MCQ-30* MetaCognition Questionnaire-30, *MUSIQOL* MUltiple Sclerosis International Quality Of Life, *PSQI* Pittsburgh Sleep Quality Index, *SDMT* Symbol Digit Modalities Test, *SEI* Self Esteem Inventory, *VAPS* Virtual Action Planning Supermarket

#### Primary outcome

The primary outcome is QoL, measured by the global score of the Multiple Sclerosis International Quality Of Life (MUSIQOL) questionnaire [17]. The MUSIQOL is a validated multi-dimensional 31-item self-report questionnaire encompassing the following nine dimensions: activity of daily living, psychological well-being, symptoms, relationships with friends and relationships with family, satisfaction with health care, sentimental and sexual life, coping, and rejection. Each item is scored on a scale ranging from 1 to 5; higher scores are suggestive of higher QoL. This questionnaire shows satisfactory psychometric properties (external validity, internal consistency, reliability, reproducibility, and acceptability), even in patients with cognitive dysfunction [18, 19]. This outcome will be evaluated during baseline and short- and long-term assessments to evaluate the immediate and long-term effect of cognitive rehabilitation, respectively.

#### Secondary outcomes

Secondary outcomes are listed as follows:

- Self-esteem measured by the Self-Esteem Inventory (SEI) [20]. This is a 58-item self-report scale. Participants must answer by ticking the box “like me” or “not like me”. The SEI is divided into four subscales: general, social, family, and professional self-esteem. The higher the score from this scale, the greater the self-esteem.
- Depression measured by the Montgomery and Åsberg Depression Rating Scale (MADRS) [21]. This is a 10-item clinician-administered scale evaluating the intensity of various depression symptoms (from apparent sadness to suicidal thoughts).
- Anxiety measured by the Hamilton Anxiety rating scale (HAMA) [22]. The HAMA consists of 14 clinician-administered items, each defined by symptoms, and measures both psychic anxiety (mental agitation and psychological distress) and somatic anxiety (physical impairments). Each item is scored on a scale ranging from 0 to 4; higher scores are suggestive of higher anxiety.
- Metacognition measured by the MetaCognitions Questionnaire (MCQ-30) [23]. The MCQ-30 is a short and easy-to-use self-reported questionnaire for multi-dimensional measure of metacognitive factors (beliefs, metacognitive judgements, and monitoring process).
- Fatigue measured by the “Echelle Modifiée d’Impact de la Fatigue dans la Sclérose En Plaques” (EMIF-SEP) (modified fatigue impact scale in multiple sclerosis [24]). This scale consists of 40 items evaluating various aspects of fatigue (cognitive, physical, and psychosocial fatigue dimensions). Each item is scored on a scale ranging from 0 (completely false) to 4 (completely true). The higher the score, the greater the fatigue.
- Subjective sleep quality measured by the Pittsburgh Sleep Quality Inventory (PSQI) [25]. This is a self-rated questionnaire which assesses sleep quality and disturbances across 19 items generating seven “component” scores: subjective sleep quality, sleep latency, sleep duration, habitual sleep efficiency, sleep disturbances, use of sleeping medication, and daytime dysfunction. The PSQI score yields a diagnostic sensitivity and specificity in distinguishing good from poor sleepers.
- Objective cognitive disorders measured by the Brief International Cognitive Assessment for Multiple Sclerosis battery (BICAMS), proposed as a brief tool for evaluating and screening cognitive disorders for neurologists, currently validated in French [26]. It consists of the Brief Visuospatial Memory Test – Revised (BVMT-R) measuring visual memory [27], the California Verbal Learning Test (CVLT) measuring verbal memory [28], and the Symbol Digit Modalities Test (SDMT) measuring information processing speed [29].

All of these outcomes will also be evaluated at all three time points: baseline (week 0), short-term retest (week 10) one week after the end of the home-based intervention, and long-term retest at week 34.

### Initial screening assessment

#### Informed consent

Before recruitment, patients will be informed about the aim, benefits, constraints, and risks of the trial. A detailed information letter and a consent form will be provided by investigators. All of the assessments at screening, baseline, and short-term and long-term visits will be conducted at the MS Expert Centre, Department of Neurology, Caen University Hospital.

#### Screening assessment

After patients have provided informed written consent, the screening visit (about 2 h) will be performed. The investigating neurologist will check inclusion and exclusion criteria mentioned above for eligibility. Then participants will undergo a neuropsychological assessment including the following:

- a cognitive complaint questionnaire
- the BCcogSEP cognitive battery (frequently used and well-validated French battery in MS derived from the Rao’s Brief Repeatable Battery of neuropsychological tests) [15, 30]
- the multiple errands test (executive functional test evaluating planning functions) [31]
- the Virtual Action Planning Supermarket (VAPS) test (virtual reality counterpart of the multiple errands test) [32]

Considering the patient’s MS clinical features and evidence of cognitive impairment from the BCcogSEP assessment, investigating neurologists and neuropsychologists will decide whether the patient is eligible or ineligible. Finally, eligible patients will be randomly assigned in the trial in accordance with the following procedure.

### Randomization and blinding

Eligible participants will be randomly assigned to one of two groups (experimental group with CR intervention or control group without CR intervention) via a computer-generated random number table in a 1:1 ratio by an independent trial-blinded statistician.

Patient, neurologist, and neuropsychologist in charge of CR intervention and psychological support for the control group will be not blinded due to the randomization procedure. The neuropsychologist in charge of clinical scales and cognitive evaluation for screening, baseline, and short- or long-term visits will be blinded.

### Duration of participant participation

Participants will be enrolled in the trial for 34 weeks, from the baseline assessment until the long-term follow-up. Assessments will be conducted with identical tools, and alternate forms of two tests (SDMT and BVMT-R) of the BICAMS were used for neuropsychological testing to reduce risk of practice effect) at baseline (week 0) and at short-term follow-up (week 10) and long-term follow-up (week 34).

### Baseline assessment

All participants will undergo a baseline assessment (duration of about 1.5 h) one week (± 7 days) after the initial screening assessment.

Baseline will be conducted by an expert psychologist and will encompass the administration of the following clinical scales and self-report questionnaires:

- MUSIQOL, which was the primary outcome measure
- SEI
- MADRS
- HAMA
- MCQ-30
- EMIF-SEP (modified fatigue impact scale in multiple sclerosis)
- PSQI

Neuropsychological assessment will be performed by using the BICAMS battery consisting of the BVMT-R, the CVLT, and the SDMT.

### Group allocation

Two neuropsychologists are involved in the trial. The first one will be in charge of CR for the intervention group and of psychological support for the active control group. The second neuropsychologist will be in charge of clinical scales and cognitive assessment for screening, baseline, and short- and long-term visits. Home-based intervention will start one week after the baseline visit and will run during eight consecutive weeks.

#### Active control group

The word “active” refers to the fact that it was not ethically possible to randomly assign patients in this group without any psychological care as some patients may be disappointed by the result of the randomization. Participants will receive only psychological support in home visits or weekly phone calls. Psychological support will consist of clinical interviews about various issues related to MS, such as identity changes, everyday cognitive-related difficulties, management of emotions, promotion of coping strategies, theoretical information about MS-related symptoms, and promotion of metacognitive awareness or cognitive strengths or both. Note that participants in the control group will be given the opportunity to have the CR program as a priority after the end of their participation.

#### Intervention (CR group)

The rehabilitation program will consist of computerized cognitive exercises from the PRESCO® software application developed by HAPPYneuron^©^ (HAPPYneuron, Inc., Lyon, France). Training sessions will consist of 19 pre-programmed exercises evaluating a broad range of cognitive domains (varying from visual memory to processing speed going through executive functioning) and were personalized for each patient (tracking tool and supervised guidance). Specifically, each task is highly flexible and can be adapted to the user to provide a customized challenge. The computer program has an automatic level progression ensuring that participants start with a level that they deem easy. This will automatically progress to a “challenge zone” where the level of difficulty will be increased to challenge participants [33].

Importantly, we previously matched pre-programmed training sessions for cognitive content, session duration, and attractive/funny dimension during a pilot trial.

At the beginning of the intervention, patients will discover the PRESCO® software application. The general procedure added with instructions and examples of pre-programmed exercises will also be shown to the patient by the referent neuropsychologist.

Then participants will undergo the cognitive rehabilitation program conducted three times per week in 45-min sessions (composed of seven exercises) during eight consecutive weeks. Patients will perform at their own pace, in the absence of the psychologist, but they may contact him at any time for technical support. They will be allowed to take breaks during sessions to manage fatigue, if present.

Participants will also receive phone calls and home visits consisting of both user-related software questions and psychological support aiming to promote coping strategies (better adjustment to current cognitive capacities). Content discussion will be the same in both groups to ensure standardization. Participants will receive feedback about their progress with the CR program.

### Short-term follow-up assessment

The short-term follow-up assessment (about 90 min) will be set once a week (± 7 days) after the end of the home-based neuropsychological management to attest to any improvement in primary and secondary outcomes.

All participants will undergo the same neuropsychological assessment as baseline assessment consisting of the BICAMS battery (SDMT, CVLT, and BVMT-R). They will also complete previously proposed scales and questionnaires (MUSIQOL, SEI, MADRS, HAMA, MCQ30, EMIF-SEP, and PSQI).

### Long-term follow-up assessment

An identical assessment will be conducted 24 weeks (± 7 days) after the short-term follow-up during which the aforementioned neuropsychological tests, scales, and questionnaires will be proposed (see details in Table 1). Adding a long-term follow-up in our trial design will allow us to detect the eventual maintenance of benefits over time in each patient’s daily life.

### Sample size and data analysis

Based on previous research [34], the standard deviation of the MUSIQOL scores was estimated at 16 points, and the correlation coefficient between scores at baseline and short-term follow-up at 0.85. We hypothesized an average increase of 3 points in the MUSIQOL scores at short-term follow-up for the active control group (a low effect size, equal to about 0.2) and an average increase of 11 points for the experimental group (0.7 effect size), leading to an 8-point difference between the two groups. Given a bilateral alpha error of 5% and a power of 80%, a minimal sample size of 18 patients per group is needed to compare the mean scores of the two groups at short-term follow-up, adjusting for the baseline scores [35]. To account for potential drop-outs, 20 patients will be included in each group.

The mean MUSIQOL scores at short-term follow-up will be compared between the two groups (CR intervention and active control) by using an analysis of covariance (ANCOVA), adjusting for the baseline MUSIQOL scores. The same type of analysis will be conducted at long-term follow-up and for secondary outcomes. An intention-to-treat analysis will be carried out as the primary analysis [36]. The results of a complementary per-protocol analysis will also be presented, as recommended by several authors [37, 38]. Significance level will be fixed at a *P* value of less than 0.05. Statistical analyses will be performed by using IBM SPSS software (IBM, Armonk, NY, USA) [39].

## Discussion

To date, pharmacological treatment has not improved or even postponed cognitive decline in MS [40]. Yet, as patients are waiting for therapeutic intervention, we must apply the most appropriate approaches in our usual health care. Such conclusions warrant prioritization of non-pharmacological, pragmatic, ecological, low-cost alternatives that address difficulties experienced in daily life [2]. As such, computer-assisted CR may bring flexibility, clinical efficacy, and ecological validity, giving an interesting clinical option for CR in the MS population [41]. In this context, we have designed an innovative home-based computer-assisted CR intervention with a specific focus on improving QoL.

The choice of this primary outcome was motivated by the paucity of existing CR trials about QoL and the need to provide psychological well-being to people with MS.

The present trial does not aim to improve cognition to a particular neuropsychological tool but rather emphasizes improvement of cognition through increased neuropsychological skills in daily living. Hence, the aim of our trial is to consider, in a real-life setting, cognitive as well as emotional and psychosocial impairment of people with MS in keeping with a holistic approach in which a patient’s feelings regarding his or her own care will be stressed.

We also decided to carry out CR by means of a computer-assisted program and a home-based intervention. Computer-assisted training offers several benefits. In our trial, exercises will be selected according to their relevance in MS as well as their level of difficulty. They will also provide immediate feedback so patients can adapt gradually and create and develop strategies increasing both QoL and cognitive functioning. A home-based CR computer program provides advantages over clinic-based training related to cost, convenience, accessibility, and transportation [3]. Patients can engage in the program without having to leave home, which can sometimes be binding for people with MS (command for an ambulance or a taxi, clinical office far away from the car park). Conversely, lack of regular one-on-one support or guidance with a professional trainer may help participants overcome cognitive disturbances and develop personal abilities by themselves. Feasibility and acceptability of a home-based computer-assisted training program were previously demonstrated in a randomized single-blind trial [3]. A main outcome aimed to specifically examine patients’ perceptions regarding home use of the program with qualitative data assessing features of the computer program, experience using the program, and strategy use [3]. The results highlighted the need to pay attention to how intervention is presented during recruitment settings and the interest of coaching and supporting the patient when using the program [3]. In most studies, intervention was presented as a “rehabilitation intervention” where participants were often closely monitored on an individual basis for some cognitive domains. However, a more pragmatic approach addressing capacities and strategies helping participants in their daily life activities may positively affect outcomes and generalization in daily life [42].

Some studies demonstrated the effectiveness to bring feedback during or after the intervention or both. They notably showed that the CR program helped patients to recognize cognitive limitations and create and practice strategies to enhance cognitive function, improving quality of their daily life [12].

Results of this trial will contribute to the limited body of literature for CR in persons with MS and provide new evidence for improving QoL and self-esteem after a tailored computer-based intervention at home. From an individual perspective, we hope to promote personal benefit (for example, better use of preserved cognitive functions for planning daily activities reducing fatigue) that patients will gain from intervention.

Our trial will have some limitations. It is a monocentric study inside an MS academic expert center with a small sample of patients. If positive, our results would be confirmed in a multicenter trial with a larger sample. The trial will concern only RR- and SP-MS patients, and the results may not be generalizable to primary progressive MS even if this form of the disease has the same pathophysiology as SP-MS and has similar cognitive impact.

If the results of this trial are positive, we anticipate making this CR program more available to interested health professionals working with people with MS as an essential part of their usual care. From a clinical perspective, the use of this home-based program, either combined with rehabilitation groups in MS care centers or alone with the support of MS clinicians, would be of interest as long as it represents an individual benefit.

## Trial status

The first participant was recruited on November 22, 2017. To date, 16 patients have been randomly assigned. The end of recruitment is scheduled in September 2020.

## Supplementary information

**Additional file 1.** SPIRIT (Standard Protocol Items: Recommendations for Interventional Trials) 2013 Checklist: Recommended items to address in a clinical trial protocol and related documents.

## Data Availability

Not applicable.

## Abbreviations

BCcogSEP: Batterie Courte d’évaluation des fonctions cognitives destinées aux patients souffrant de Sclérose En Plaques (French brief test battery evaluating cognitive functions in multiple sclerosis)
BICAMS: Brief International Cognitive Assessment for Multiple Sclerosis
BVMT-R: Brief Visuospatial Memory Test – Revised
CR: Cognitive Rehabilitation
CVLT: California Verbal Learning Test
EDSS: Expanded Disability Status Scale
EMIF-SEP: Echelle Modifiée d’Impact de la Fatigue dans la Sclérose En Plaques (modified fatigue impact scale – multiple sclerosis)
HAMA: Hamilton Anxiety rating scale
MADRS: Montgomery and Åsberg Depression Rating Scale
MCQ-30: MetaCognition Questionnaire-30
MS: Multiple sclerosis
MUSIQOL: MUltiple Sclerosis International Quality Of Life
PSQI: Pittsburgh Sleep Quality Index
QoL: Quality of life
RR: Relapsing-remitting
SDMT: Symbol Digit Modalities Test
SEI: Self-Esteem Inventory
SP: Secondary progressive
